# Supplementary material for: Are carbon emissions trading and green financial instruments synergistic? -Comprehensive quantitative research based on content analysis
Source: PLoS One. 2024 Mar 7;19(3):e0298601. doi: 10.1371/journal.pone.0298601 (PMC10919601; doi:10.1371/journal.pone.0298601)
Supplement: S1 Data — (ZIP) [file pone.0298601.s001.zip › Data/地级市Z_F工作报告.zip!/║■▒▒/╩«╤▀╩╨/└┤╫╘╣1⁄2╓┌║┼í╛┬φ┐╦╩2╛▌═°í┐_2011.docx]

各位代表：

　　现在，我代表市人民政府向大会报告政府工作，请各位代表连同《十堰市国民经济和社会发展第十二个五年规划纲要（草案）》一并审议，并请各位政协委员和列席人员提出意见。

　　一、过去六年政府工作的回顾

　　本届政府从2005年开始履职，已经走过整整六年时光。六年的历程，在十堰发展史上极不平凡。面对东风公司“两个总部”外迁、国际金融危机冲击、自然灾害频发等多种困难和挑战，在市委的正确领导下，全市上下团结一心，克难奋进，全面加快小康社会建设，奋力打造区域性中心城市，经济社会发展取得了巨大成就，提前一年完成了“十一五”规划的主要目标任务，为长远可持续发展奠定了坚实基础。

　　（一）经济实力大幅提升。

　　预计，2010年全市实现地区生产总值736亿元，比上年增长24.2%，是2004年的2.1倍，年均增长13.3%。地方一般预算收入43.8亿元，比上年增长69.9%（扣除不可比因素增长27.9%），是2004年的4.3倍，年均增长27.6%。全社会固定资产投资406亿元，比上年增长46%，是2004年的5.2倍，年均增长31.8%。

　　农村经济稳步发展。2010年实现县域生产总值295亿元，年均增长10.3%；劳务经济总收入51.9亿元，年均增长16.3%。农业产业化加速推进，六年新建特色产业基地120万亩，新增市级以上龙头企业115家。农民生产生活条件显著改善，累计建设、改造通村水泥路1.3万公里，解决98.8万人安全饮水问题，新建农村户用沼气13.3万户。新阶段扶贫开发整村推进任务基本完成，减少贫困人口43万。

　　工业经济实现历史性突破。2010年全市工业总产值突破千亿元大关，完成1313亿元，年均增长18.2%；规模以上企业近千家，产值规模过亿元的企业达130家；地方工业比重超过50%。项目建设成效显著，东风渝安20万辆扩能、东风实业3万辆整车、正和车身3万辆等重大项目建成投产。汽车产业增势强劲，实现产值973.9亿元，整车产量达到56.2万辆，十堰荣膺“中国卡车之都”称号。开发区土地集约、企业集中、产业集聚的优势进一步显现，全市开发区工业产值达到375亿元。

　　第三产业持续繁荣。2010年全市社会消费品零售总额实现305亿元，比上年增长20%，年均增长18.5%。旅游产业持续高速发展，接待国内外游客1477万人次，实现旅游收入89.3亿元，年均分别增长19.7%和26.9%；3A级以上景区由2004年的1家增加到25家，武当山通过5A级景区验收。现代金融服务能力不断增强，金融机构各项存款余额达到857.6亿元，年均增长23.3%；各项贷款余额为410.3亿元，年均增长28.4%。新近引入的兴业银行已在十堰正式挂牌营业。

　　（二）基础设施建设明显提速。

　　交通和水利水电建设成效卓著。累计完成交通基础设施建设投资219.1亿元，全市公路通车总里程达2万公里。武当山机场通过预可研评估；襄渝铁路二线十堰段建成通车，武汉至十堰动车组全线开通；福银高速十堰段、武白一级路、武神路竣工通车，十白、十房、谷竹高速和郧十一级路加快推进，郧十高速、东环路开工建设，十堰市正式被确立为全国公路运输枢纽城市。全市累计新增水电装机35.4万千瓦，南水北调控制性项目丹江大坝加高工程顺利完工。

　　城市功能更趋完善。新一轮城市总体规划和土地利用总体规划修编全面完成，城市空间拓展了近30平方公里。节约集约，创新模式，整理山地5万多亩，十堰市的经验现已在全国推广。新建天津路、重庆路、浙江路、上海路、东风大道、凯旋大道等城市主干道，完成城区9条道路和108条内街小巷改造建设，改造图书馆、人民广场、人民公园，建成博物馆、游泳馆、赛武当广场等一批市政公共设施。市县城镇建设步伐加快，城镇化率已达41%。

　　（三）发展动力进一步激活。

　　重点领域和关键环节改革深入推进。国有企业改制改革基本完成。农村综合配套改革和集体林权主体改革顺利完成。医药卫生体制改革全面启动。新一轮政府机构改革全面完成。行政审批制度改革继续深化，减少行政审批361项，减幅达40%以上。省管县财政体制改革推进到位，部门预算和国库集中支付实现全覆盖。

　　外向型经济不断壮大。2010年全市实际利用外资7203万美元，比上年增长25.6%，年均增长11%；利用市外境内资金96亿元，年均增长26.9%；外贸出口2.2亿美元，比上年增长16.2%，年均增长30.2%。

　　两届武术节成效斐然。举全市之力，聚全民之智，办大事、办难事，高标准、高质量成功举办第三、第四届世界传统武术节，取得了赛事规模和竞技水平的新突破，赢得了海内外的广泛赞誉。通过办节办会，提升了城市品位，展示了十堰形象，扩大了对外交流，进一步凝聚了人心，提振了士气，增强了城市活力。

　　（四）民生事业持续改善。

　　科教文卫事业协调发展。累计实施重点科技专项300多个，新增高新技术企业10家。改扩建危房校舍86.7万平方米，建成农村寄宿学校667所，创建省级以上示范学校8所，十堰职业技术学院、郧阳师范高等专科学校顺利搬迁。改扩建医疗卫生机构878个，新型农村合作医疗参合率提高到96.2%，区域性公共医疗服务中心基本形成。新建、改造农村各类文体设施2069个，“武当神戏”等7个非物质文化遗产入选国家保护名录，广播电视综合人口覆盖率达94%，群众性文体活动日益丰富。

　　就业和社会保障水平进一步提高。累计新增城镇就业20.5万人，安置下岗失业人员9万余人。五项社会保险参保人数达190万人；城市低保实现应保尽保，农村低保基本实现全覆盖，55%的农村五保户实现集中供养。发放住房公积金贷款15.6亿元、困难家庭租赁补贴4611万元，建成廉租住房和经济适用房近万套。

　　城乡居民收入稳步增长。2010年城镇居民人均可支配收入12652元，比上年增长11.2%，年均增长11.2%；农民人均纯收入3499元，比上年增长12.5%，年均增长10.6%。

　　南水北调移民外迁任务圆满完成。全市上下排除万难、合力攻坚，在不到一年时间内集中外迁7.6万移民，实现了平安搬迁、有序搬迁、和谐搬迁，创造了水库移民史上的奇迹。

　　社会大局和谐稳定。建立健全社会治安综合治理长效机制，妥善处置信访积案、难案，及时排查化解了社会矛盾。全面落实安全生产责任制，亿元生产总值死亡率连续六年持续下降。

　　（五）政治文明和精神文明建设扎实推进。

　　民主法制建设取得新的进展。坚持定期向人大报告工作、向政协通报情况，重大决策及时向民主党派、无党派人士和人民团体征求意见，累计办理办结人大代表议案、意见、建议1095件，办理办结政协委员提案2198件，办结满意和基本满意率分别达到98.9%和98.4%。全面完成了“五五”普法工作，扎实开展了政风行风评议。

　　精神文明建设取得新的成效。十堰市连续两届荣获全国创建文明城市工作先进城市称号，连续五届获得省级文明城市称号，国防动员和民兵预备役等工作取得明显成绩，蝉联全国双拥模范城“五连冠”、省级双拥模范城“六连冠”。竹山县城、丹江口市习家店镇和郧西县湖北口回族乡、市太和医院分别被授予全国文明县城、全国文明村镇和全国文明单位荣誉称号。

　　（六）生态环境建设成效显著。

　　累计完成人工造林279万亩，国家和省级自然保护区面积达到335万亩，森林覆盖率提高到53%；综合治理小流域307条，治理水土流失面积2700平方公里；建成10座污水处理厂和5座垃圾处理厂；关闭污染企业300多家，治理重点排污企业86家，主要污染物排放量提前一年达到排放目标。丹江口水库水质优于国家Ⅱ类标准。单位生产总值能耗下降20%。

　　各位代表！六年来，我们始终坚持把改善民生作为第一责任，把群众满意作为第一标准，千方百计、真情实意为民办实事、办好事、解难事，圆满完成了历年的“十件实事”，解决了一批社会高度关注、群众反映强烈的热点难点问题。

　　刚刚过去的2010年，在市委的总体安排部署下，我们成功举办了第四届世界传统武术节，圆满完成南水北调移民外迁任务，精心谋划“十二五”发展蓝图，顺利通过创卫省级评估，全面启动竹房城镇带城乡一体化试验区建设，经济提质提速，民生持续改善，社会稳定和谐，超额完成全年主要目标任务。

　　在经济社会快速发展的过程中，全市民族宗教、外事、侨务、对台、司法、审计、监察、统计、气象、水文、地震、人防、老龄、残联、社科、档案、地方志等工作也都取得了新的成绩。

　　各位代表！六年的成绩来之不易，发展的成果振奋人心，积累的经验弥足珍贵。十堰已度过了最为困难的时期，进入了厚积薄发的新阶段，站在了新的历史起点上。这些成就的取得，是在市委的正确领导下，历届政府励精图治、不懈奋斗的结果，是市人大、市政协加强监督、大力支持的结果，更是全市人民和衷共济、团结拼搏的结果。在此，我代表市三届人民政府，向所有参与、支持、关心十堰发展的同志们、朋友们以及社会各界人士表示衷心的感谢并致以崇高的敬意！[FS:PAGE]

　　二、“十二五”时期经济社会发展的指导思想、基本原则和主要任务

　　“十二五”时期，是十堰推进跨越式发展的战略机遇期，我们面临诸多难得的历史机遇：国家实施扩大内需和促进中部地区崛起战略、实施新一轮西部大开发和扶贫开发，全省致力于打造鄂西生态文化旅游圈、建设竹房城镇带城乡一体化试验区，东风公司全面实施“61行动计划”、政企共建百万量级汽车城，市内一大批重大基础设施和生产性项目相继开工建设，全市加快科学发展的环境更优、条件更好、基础更实。在看到机遇的同时，我们也清醒地认识到，经济社会发展中仍然存在不少困难和挑战：产业结构不尽合理，培育新的支柱产业迫在眉睫；县域经济总量不大、实力不强，农民持续增收的难度加大；城乡二元结构矛盾依然突出，统筹城乡协调发展任重道远；劳动就业、社会保障和移民内安后靠等任务繁重，改善民生、维护稳定的压力加大；生态建设和环境保护的硬约束增强，转型发展还需有大的突破；面对周边地区强劲发展的态势，十堰市面临前所未有的竞争压力；少数干部作风漂浮、工作不实、效率不高，与快速发展的全局态势还不相适应。有效应对挑战，切实维护好当前难得的经济生态、政治生态、社会生态和自然生态，必须更加奋发有为，推进跨越式发展。

　　“十二五”时期全市经济社会发展的指导思想是：高举中国特色社会主义伟大旗帜，以邓小平理论和“三个代表”重要思想为指导，深入贯彻落实科学发展观，以科学发展为主题，以加快转变经济发展方式为主线，以推进新型工业化、新型城镇化和农业现代化为重点，以解放思想、改革开放为动力，以提高城乡人民生活水平为根本出发点和落脚点，加快推进“一城两带”建设，努力实现十堰经济社会跨越式发展，为全面建设小康社会打下更加坚实的基础。

　　基于以上指导思想，我们将着重把握以下原则：坚持抢抓机遇、加快发展，抓住一切有利的政策机遇，坚定不移地把加快发展作为第一要务，不断增强综合竞争实力；坚持转变方式、转型发展，在发展中促转型，在转型中谋发展，加快构建现代产业体系，促进经济增长由主要依靠投资拉动逐步转向依靠消费、投资、出口的协调拉动；坚持统筹城乡、协调发展，统筹城乡空间布局、产业发展、基础设施建设、社会事业发展，加快城乡一体化进程；坚持改革开放、创新发展，把深化改革、扩大开放作为发展的根本动力，不断增强自主创新、自我发展能力；坚持生态环保、绿色发展，把建设两型社会放在更加突出的位置，加快探索适合十堰市情的可持续发展之路；坚持改善民生、共享发展，更加注重民生工作和社会建设，加快社会保障体系建设，促进公共服务均等化。

　　综合考虑未来发展的趋势和条件，今后五年发展的主要目标是：力争开创区域性中心城市建设、综合交通枢纽建设、城乡一体化发展、山水一体大旅游和生态文明建设的新局面。主要经济指标力争实现翻番，地区生产总值年均增长12%以上，地方一般预算收入年均增长15%，全社会固定资产投资年均增长20%，社会消费品零售总额年均增长20%，规模以上工业增加值年均增长15%，利用外资年均增长15%，外贸出口年均增长18%，城镇居民人均可支配收入和农村居民人均纯收入年均分别增长12%以上，高新技术产业增加值占生产总值比重达到15%以上，单位生产总值能耗下降、单位生产总值二氧化碳、主要污染物排放控制在省定目标以内。

　　围绕上述指导思想、基本原则和主要目标，“十二五”期间将重点做好以下八个方面的工作：

　　（一）坚持以“一城两带”为总体战略，大力促进区域协调发展。

　　加快十堰城区建设。充分发挥武当山、丹江水、汽车城品牌效应，打造国际知名生态文化旅游区、国家级汽车产业集聚区、国家级生态经济示范区和区域性现代服务中心。加快市县对接步伐，完善公共服务体系，提高城市建设管理水平，增强发展动力和活力。到2015年，力争在集约节约的基础上，建成区面积达到100平方公里，城区基础设施人口承载能力超过100万，汽车产能达到百万量级规模，基本建成区域性中心城市。

　　加快竹房城镇带城乡一体化试验区建设。创新体制机制，统筹推进城乡规划布局、产业布局、基础设施、社会事业、公共服务和生态建设，力争将实验区建成全省山区新农村建设的引领区、城乡一体化的先行区和统筹城乡发展的示范区。

　　加快汉江生态经济带建设。统筹推进汉江沿线城乡空间布局、产业发展、移民安置、生态建设、基础设施建设和社会事业发展，逐步将该区域建设成为生态优良、经济发达、城乡协调、生活富裕的生态文明示范区。

　　（二）坚持以转变发展方式为主线，全力推进产业结构优化升级。

　　做强做大主导产业。全力支持服务东风公司等重点企业，将十堰打造成为国内最具规模、最具实力的商用车制造基地，最重要的微型车、专用车、汽车零部件及汽车装备生产基地。全面提升地方汽配产业自主发展能力，打造国家级汽车产业集聚区、商用车零部件产业集群创新示范区，力争十堰经济开发区升级为国家级经济技术开发区。

　　加快发展以旅游业为先导的现代服务业。放大武当山龙头效应，加快太极湖新区建设，深度挖掘道教文化、汉水文化、汽车文化等特色文化资源，强化鄂西生态文化旅游圈核心板块和重要支撑的地位。完善生产性服务业，发展生活性服务业。

　　进一步加快水电、绿色食品和生物医药产业发展。积极扶持电子信息产业和现代制造业发展。加快培育节能环保、新能源、新材料、新能源汽车等战略性新兴产业。

　　（三）坚持以“一统三分”为基本方略，扎实推进新农村建设。

　　按照农业抓产业、农民抓就业、农村抓事业的思路，以农民长效增收为核心，着力发展高效生态绿色农业，加快农业产业化进程，努力实现农村经济新跨越。

　　大力发展山区特色现代农业。进一步扩大特色产业基地规模，力争基地面积达500万亩。突破性发展农产品深加工，加速培育一批全国、全省有影响的龙头企业和知名品牌，实现加工产值200亿元以上。积极发展设施农业，加快实施新一轮“菜篮子”工程，提高蔬菜等生活必需品的自我供给能力。深入推进农业机械化和标准化建设，不断提高农业物资装备水平。加强农产品质量安全监管。促进农村合作经济组织发展。

　　千方百计拓宽增收渠道。加大农村剩余劳动力培训力度，加快农村人口向城镇转移速度，切实提高劳务经济水平，力争转移农村剩余劳动力100万人以上，实现劳务收入100亿元以上。积极推进乡村旅游富民工程，扶持规范农家乐发展。探索建立土地、房产、林权等农村资产要素市场化机制，逐步增加农民资产流转收入。

　　加快以水利为重点的农村基础设施建设。解决86万农村人口安全饮水问题，新建10万户农村户用沼气，除险加固病险水库272座，新增灌溉面积17万亩，改善灌溉面积35万亩，新建农村水泥（沥青）路1万公里。

　　深入推进扶贫开发。抓好丹江口市整市推进脱贫奔小康、竹山县和郧县连片开发试点工作，启动新一轮整村推进扶贫开发，完成新一轮城区农村扶贫规划，减少农村贫困人口50%以上。

　　（四）坚持以重大项目建设为支撑，努力集聚发展后劲。

　　加快构建立体交通网络。建成武当山机场，力争十宜、十三铁路全线开工建设，建设两个码头、四大港口，全面升级国省道干线公路，建成市域内2小时交通经济圈，构建内畅外联、辐射全国、换乘便捷的综合交通运输网络。

　　加快能源基地建设。潘口、三里坪、龙背湾、孤山等水电站和热电联产项目建成投产，争取国家、省在十堰市规划布点路口火电项目，同步抓好骨干电源的输变电网络建设。力争“十二五”期间新增水电装机100万千瓦。

　　加快市政设施建设。加速城市道路、公交、污水处理厂、垃圾处理厂、天然气等公共设施建设，全力改善城区交通拥堵状况，注重开发利用城市地下空间，推进“三网融合”，建设“数字十堰”，力争建成全国文明城市、国家卫生城市、国家环保模范城市。

　　（五）坚持以改革开放为动力，进一步增强发展活力。

　　深化经济体制改革。积极构建有利于转变经济发展方式的财税体制，健全服务社会、惠及民生的公共财政体系。大力引进民营股份制银行，创新农村金融服务，确保企业上市工作取得实质进展。进一步建立完善国有资产监管体制，实现国有资产保值增值。

　　加快社会管理体制改革。积极稳妥推进事业单位分类改革，全面推行事业单位绩效工资制度。培育扶持和依法管理新经济组织和新社会组织，支持、引导其参与社会管理和服务。改革基本公共服务供给方式，推进非基本公共服务市场化改革，满足群众多样化需求。

　　全力以赴招商引资。大力营造招商、亲商、安商、利商的良好氛围，进一步建立完善招商引资激励机制，鼓励民间扩大投资，积极引导全民创业，加速培植更多的市场主体。

　　努力扩大外贸出口。加快出口平台建设，进一步扩大优势产品和特色农副产品出口，加速国家级汽车及零部件出口基地建设，积极培育新的外贸增长点。

　　（六）坚持以生态文明建设为立足点，加快两型社会建设。

　　加强生态保护。切实抓好南水北调中线核心水源区生态建设，确保一江清水入库、一库清水永续北送；扎实推进退耕还林、天然林保护、库周生态隔离带等工程建设；全市森林覆盖率提高到58%以上。积极探索建立生态补偿、对口支援等双赢机制，全力争创国家“生态文明建设综合配套改革试验区”。

　　大力推进节能减排。严格实行污染物达标排放和总量控制，重点抓好工业、交通、建筑等领域的节能减排，有效控制点源、面源污染，实现单位生产总值能耗下降16%，城市污水集中处理率和生活垃圾无害化处理率均达到95%以上。

　　（七）坚持以改善民生为根本，切实提高公共服务保障能力。

　　加快教育和人才事业发展。大力推进教育机制创新，加大投入，优化布局，加快普及学前教育，促进义务教育均衡发展，逐步普及高中阶段教育，建立特色职业教育和区域高等教育体系。深入推进人才强市战略，优化人力资源，构筑区域人才高地。

　　大力发展卫生和文体事业。加强公共卫生服务体系建设，加快推进医药卫生体制改革，稳妥推进公立医院改革。完善公共文化体系，深化文化事业单位机制改革，大力推动经营性文化单位转企改制，加速发展文化产业，完成十堰日报传媒集团、广电传媒集团和十堰大剧院建设，力争文化产业增加值年均增长20%，满足人民多元化文化需求。加大公共体育设施建设力度，大力开展全民健身活动。

　　做好人口计生工作。坚持计划生育基本国策，坚持控制人口总量与提升人口素质并重、改善人口结构与优化人口布局并举，统筹解决人口问题，促进人口长期均衡发展。

　　提高就业和社会保障水平。重点解决农村剩余劳动力、高校毕业生、困难人员就业问题，积极实施“回归创业工程”，确保新增就业20万人次。力争五项社会保险参保人数达230万人次，城乡低保补助标准年均增长20%，农村五保自愿集中供养率达80%以上。扩大住房公积金覆盖面，加大保障性安居工程建设力度。积极发展妇女儿童和老龄服务事业。

　　（八）坚持以安全稳定为前提，着力维护社会和谐。

　　加大社会矛盾排查化解力度。畅通和规范群众诉求表达、利益协调、权益保障渠道，进一步完善联合接访机制，重心下移，力争把各种不稳定因素化解在萌芽状态。全面落实各项移民扶持政策，确保安全有序完成移民内安及搬迁复建任务，统筹谋划安置区长远发展。

　　强化安全生产监管。提升安全生产保障能力，健全事故灾难、公共卫生事件、食品安全事件、社会安全事件的预防预警和应急处置体系，坚决遏制重特大事故发生。

　　加强社会治安综合治理。完善社会治安防控体系，严密防范和严厉打击各种违法犯罪活动，切实保障人民群众生命财产安全。

　　切实加强国防建设。支持驻军和武警部队建设，抓好国防动员、民兵预备役、人民防空等工作。落实军人军属优抚安置政策，深入开展双拥共建活动，巩固发展军政军民团结。[FS:PAGE]

　　三、扎实做好“十二五”开局之年的工作

　　今年是“十二五”规划实施的第一年。开好局、起好步，对全面完成“十二五”规划目标任务、实现跨越式发展至关重要。

　　今年政府工作的总体要求是：认真贯彻落实市委三届八次全会和全市经济工作会议精神，突出主题，围绕主线，全面提升主导产业，培植壮大优势产业，全力推进县域经济，深化改革，扩大开放，建设生态文明，改善保障民生，促进社会和谐，为十堰跨越式发展打下坚实基础。为此，我们将坚持把更好更快发展作为首要任务，努力做到好中求快、力攀新高；坚持把调结构、促转型作为主攻方向，努力提高经济增长的质量和效益；坚持把保障和改善民生作为加快发展的根本出发点和落脚点，努力让人民群众共享改革发展成果；坚持把转变作风、真抓实干作为环境建设的重中之重，努力提高推进经济社会跨越式发展的保障能力。

　　今年全市经济社会发展的主要预期目标是：地区生产总值增长13%；地方一般预算收入增长15%；全社会固定资产投资增长25%以上；社会消费品零售总额增长20%以上；规模以上工业增加值增长18%；实际利用外资增长12%，外贸出口总额增长15%；城镇居民人均可支配收入和农民人均纯收入分别增长12%以上；城镇新增就业3.5万人，城镇登记失业率控制在4.5%以内；居民消费价格指数控制在4%左右；人口自然增长率、单位生产总值能耗下降、主要污染物排放量控制在省定目标以内。

　　按照上述要求和目标，我们将突出抓好事关全局的六项重点工作：

　　（一）全面提升主导产业，进一步增强核心竞争力。

　　围绕建设百万量级汽车城目标，全力支持东风公司实施“61行动计划”，大力扶持有实力的企业加快发展，坚持整车与总成同步、生产与研发并举，集聚要素，整合资源，放大商用车比较优势，确保汽车工业产值突破1000亿元。

　　盘活存量，培育增量，大力发展整车和专用车。支持东风商用车联合工厂一期4万辆、三环专汽3万辆扩能项目建成投产，启动东风渝安三期10万辆整车项目建设；力争东风征梦3万辆、安远2万辆、驰田2万辆、东风特汽1万辆、神河1万辆等专用车项目建成投产。实现整车产销60万辆以上、专用车产销10万辆以上。

　　全面发展5大总成，做专做精零部件和装备产业。支持东风18万台发动机、18万套变速箱动力总成项目建设，确保东风特种车身15万辆、正和车身10万辆扩能项目开工。力争双星东风千万套级轮胎扩能项目建成投产，支持先锋模具、佳恒液压、华昌达等装备企业快速发展。进一步扩大汽车汽配会展经济影响力，增强中国（十堰）汽配城辐射能力。

　　全力支持地方企业发展，不断增强企业创新能力。加快推进地方专用车生产企业与东风商用车公司合作发展，提升地方企业竞争力。推进创新型企业建设，积极申报国家数字化装备重大专项应用示范工程，加速推进国家汽车质检中心零部件分中心建设。

　　以现有零部件企业和装备企业为基础，既立足于汽车产业又着眼于超越现有产业，加快发展先进制造业。着力推进企业技术改造和技术创新，通过技术结构调整推动产品结构调整，通过产品结构调整推动企业结构调整，通过企业结构调整推动整个产业结构调整，提升产业层次。大力延伸产业链条，千方百计开拓汽车产业以外的配套领域，切实增强配套产业自主发展的内生动力。

　　（二）突破性发展旅游服务业，全力培植新兴支柱产业。

　　旅游服务业是当今世界发展最快的新兴产业。十堰旅游资源得天独厚、禀赋极高，发展旅游业空间和潜力巨大。要站在全局和战略的高度，把质变蕴于量变之中，在量变中酝酿质的飞跃，创新理念提升旅游，政府主导大办旅游，市场营销拉动旅游，打造品牌做强旅游，力争全年接待游客1800万人次以上，旅游总收入突破110亿元。

　　以武当山为龙头，以太极湖为引擎，全力打造旅游核心区，确保太极湖新区重点项目全部开工，基本完成武当新区、水上游览区、休闲区三大板块的主体工程；加快建设玉虚宫、五龙宫、遇真宫修复等项目，建成武当山旅游码头。创意发展一批道教养生、道教音乐、古建筑鉴赏等体验式项目，对接发展一批乡村游、生态游、养生游联动项目，配套发展一批餐饮、住宿、娱乐、购物服务项目，提高旅游产业综合效益。不断壮大市场主体，培育引进一批有竞争实力的旅游骨干企业，推进旅游业超常规发展。全力破解融资难题，加快旅游投资公司建设，创造条件推进企业直接向资本市场融资。强化旅游行业管理，积极培育和引进旅游高端人才。加大宣传促销力度，让“问道武当山、养生太极湖”主题形象世人皆知。努力推进旅游由单纯观光向观光、休闲、养生并重延伸，由单一资源主导向资源与文化并举转变，由门票经济向产业经济转型。

　　（三）强力助推县域经济，加快城乡协调发展步伐。

　　强市之基在于强县，富民之源在于富县。十堰要实现跨越式发展，县域经济必须加快实现跨越式发展。要抢抓全省县域经济工作会议在十堰市召开的契机，坚持用工业化理念谋划农业，用城镇化理念建设农村，扎实抓好“三农”工作，全力助推县域经济发展，力争各县市在全省县域经济综合排名中的位次都有新的提升。

　　大力培育市场主体。坚持“产业第一、企业家老大”的理念，做大主导产业，做强骨干企业，引进实力企业，提升本土企业。坚持差异化发展策略，突出“一县一品”，壮大水电能源、机械制造、生物医药等产业，力争各县市新增规模企业10家以上。大力开展全民招商、专业招商、大员招商，扩大招商领域，提高招商实效。

　　着力构建发展平台。按照“特色鲜明、优势互补、错位竞争、差异发展”的原则，坚持政策向园区倾斜、企业向园区集中、产业向园区集聚，加快园区建设步伐。坚持规划先行、科学定位，集约节约利用土地，高质高效发展园区。加快县市融资平台建设，构建政府主导、市场运作、社会参与的多元化投融资机制。今年市政府将把融资平台建设纳入各县市目标考核，并通过以奖代补方式予以支持。

　　加快发展农业产业化。进一步壮大特色产业基地，新建基地10万亩，改造升级15万亩，新建畜牧养殖小区120个。大力推进农产品规模化生产、标准化加工、商品化包装、品牌化推介、市场化营销，重点支持一批龙头企业做强做大，力争新增市级以上龙头企业20家，实现加工产值65亿元以上。加强“武当道茶”、丹江鲌鱼等品牌建设，确保新认证“三品”品牌、地理标志产品和知名商标20个，力争“武当道茶”成为中国驰名商标。新建、改造蔬菜基地各1.5万亩。按照今年中央1号文件的要求，把水利作为农村基础设施建设的重点，加大投入，加快发展。同步推进基本农田、农村能源、乡村公路等基础设施建设，进一步改善农村生产生活条件。

　　加速推进城乡一体化。按照3年取得明显变化、5年基本建成的目标，加快建设竹房城镇带。坚持规划先行，坚持区域统筹，坚持产业主导，坚持改善民生，坚持改革创新，坚持群众主体，尽快启动一批产业发展、基础设施和村庄整治项目，今年力求取得实质性进展。按照生态与经济协调发展的要求，加快汉江生态经济带建设，力争将其提升到更高的决策层面。继续加快7个省级、1个市级新农村示范乡镇建设和丹江口市整市推进脱贫奔小康试点工作。

　　（四）加强重大基础设施建设，夯实跨越式发展的基础。

　　强力推进交通项目建设。力争武当山机场开工建设，加快谷竹、十白、十房和郧十高速公路建设，积极争取国省干线公路调整项目，基本建成郧十一级公路。

　　加快市政设施建设。确保东环路建成通车、发展大道路基工程完工，推进中岳路建设，启动车城西路改造和林荫大道建设工程。建成东部垃圾处理厂和西部污水处理厂，完成天然气十堰支干线及城网配套工程。抓好城市综合改造和专项整治，加快城区综合配套设施建设，依法查处违法违章建筑，力争全国文明城市和国家环保模范城市创建能进入申报程序、国家卫生城市创建通过评估。

　　（五）着力保障和改善民生，让发展成果惠及更多百姓。

　　全面发展社会事业。积极发展学前教育，均衡发展义务教育，特色发展高中教育，大力发展职业教育，加快中小学校舍安全工程建设，支持十堰职业技术学院创办国家骨干职业院校，支持郧阳师专创建省学前教育示范学校，支持武当山国际武术学院秋季建成招生。巩固基本药物制度改革试点成果，抓好扩大试点工作，力争政府举办的基层医疗卫生机构全部实施基本药物制度，确保红十字医院改革试点取得实质性进展，大力推行就医“一卡通”，稳定提高新型农村合作医疗参合率。进一步完善城乡文体设施，推进山地体育公园建设，继续实施广播电视“村村通”工程，丰富群众文体生活。

　　加强就业和社会保障工作。把促进充分就业作为优先目标，扎实推进创业就业，确保新增城镇就业3.6万人、再就业1.7万人，培训转移农村劳动力3万人。进一步扩大社会保险覆盖面，确保扩面新增6万人；积极推进城乡低保一体化试点；做好新型农村养老保险改革试点工作；加强福利院、光荣院和老年公寓建设。

　　加强和创新社会管理。明确社区事权，按照财权、事权对等原则，进一步加大经费保障力度，完善社区服务功能，提升社区管理水平。

　　讲政治，顾大局，把移民安置作为头号民生工程来抓。基本完成南水北调10万移民内安迁建任务，全面完成潘口水电站移民安置任务，妥善解决移民内安及搬迁复建中出现的矛盾和问题。

　　把稳控物价放在更加突出的位置，坚持自我生产和外部调运两手抓，保障市场供给，强化市场监管，保持市场价格总水平基本稳定。认真贯彻国家房地产宏观调控政策，有效抑制房价过快上涨，实行保障性住房与商品性住房双管齐下、双线运行，加快以公租房为重点的保障性住房建设，满足中低收入家庭住房需求。进一步完善信访工作责任制，健全社会舆情汇集和分析机制，健全社会稳定风险评估机制，下功夫从源头上化解矛盾和问题。同时，深入开展平安创建活动，加强安全生产和社会治安综合治理，确保人民生命财产安全。

　　（六）扎实办好“十件实事”，认真解决好事关群众切身利益的问题。

　　各位代表！民生连着民心，民心凝聚民力。今年，市政府将突出重点，加大投入，完善措施，继续办好城乡群众普遍关心的十件实事。（1）解决农村10万人安全饮水问题。（2）新建300个农村村级综合服务社、新建改造20个社区综合服务站。（3）完善26个乡镇卫生院医疗急救站。（4）改造社区公厕和设置生态移动公厕30个。（5）改造17个老式垃圾中转站。（6）维修改造城区60条内街小巷。（7）新建30个公交站廊。（8）全程改造美化城区人民路。（9）启动建设1000套公共租赁住房。（10）新建和改建5所幼儿园。[FS:PAGE]

　　四、努力建设人民满意的政府

　　让人民满意是政府工作的不懈追求。我们将在市委正确领导下，牢记宗旨，以人为本，依法行政、以德施政、科学理政、廉洁从政，全力打造法治政府、效能政府和阳光政府。

　　（一）推进依法行政，着力打造法治政府。

　　依法行政是政府一切工作的行为准则。我们将严格依照宪法和法律规定的权限和程序，全面履行经济调节、市场监管、社会管理和公共服务职能，促进行政行为规范化，确保行政行为合法化，严格实施依法治市。

　　提高公务人员法律素养是依法行政的前提。我们将广泛开展“法律进机关”活动，狠抓普法教育和岗位培训，不断增强公务人员法治理念，切实提高依法办事的操作能力和依法行政的服务水平。

　　加强行政决策制度建设。逐步建成公众参与、专家论证、风险评估、合法性审查和集体讨论决定相结合的决策机制，探索建立重大行政决策实施情况民意调查和社会效果评价制度，及时发现并弥补行政决策中的不足，扎实推进行政决策的科学化、民主化、法治化进程。

　　规范行政执法行为。按照规范执法、公正执法、文明执法的要求，规范执法主体，落实执法人员持证上岗和资格管理制度。完善执法程序，做到流程清楚、要求具体、期限明确。改进执法方式，平等对待行政相对人，维护相对人人身权利和人格尊严。

　　积极接受监督。主动接受人大、政协监督，强化上级行政机关对下级行政机关的监督，拓宽群众监督渠道，自觉接受社会监督，高度重视舆论监督，依法规范政府行为。

　　（二）提高执行能力，着力打造效能政府。

　　执行力是效能建设的核心。要加强作风建设，增强执行能力，努力实现公平高效、勤政高效、廉政高效、善政高效、低耗高效，切实提高政府效能。

　　推进机制创新。完善主体明确、层级清晰、具体量化的岗位责任制，实现责任无缝链接。探索绩效评估制度，科学设置指标体系，推进绩效评估制度化。完善重大事项协调调度制度，确保政府各项工作指挥有方、推进有序、落实有力。

　　优化审批流程。创新工作运行程序和方式，加快构建“一楼式办公”、“一窗式收费”、“一站式服务”的行政服务体系，避免多头审批、重复审批。强化行政服务中心职能，探索推进“三集中、三到位”行政审批相对集中改革，提高行政审批效率。推行网上电子审批，构筑高效便捷的服务平台。

　　切实改进政风。始终保持昂扬向上、奋发有为的精神状态，始终保持迎难而上、知难而进的旺盛斗志，始终保持同人民群众的血肉联系。严明纪律，服从大局，确保政令畅通，做到定必干、干必成、成必优。以对党和对人民高度负责的精神，尽职尽责做好各项工作，努力形成一级抓一级、层层抓落实的良好局面。下大力气精简会议、压缩文件，认真开展调查研究，集中精力抓大事、抓要事、抓难事。

　　严格行政问责。坚持有错必纠、有责必问，严格落实首问负责、限时办结、责任追究三项制度，杜绝有令不行、有禁不止、行政不作为、失职渎职等行为发生。

　　（三）坚持行政为民，着力打造阳光政府。

　　阳光施政是现代政府的重要特征。要让政务在阳光下操作，权力在阳光下运行，努力塑造公开透明、便民利民、清政廉洁的政府形象。

　　全面推进政务公开。以公开为原则、不公开为例外，凡是不涉及国家秘密、商业秘密和个人隐私的政府信息，都及时向社会公开。进一步完善重大决策听证、重要事项公示、重点工作通报、政务信息查询“四项制度”，着重抓好政府文件、决策过程、政务活动、办事程序、收费标准、处理结果“六个公开”，实现公共权力透明行使、公共资金透明运行、公共资源透明分配、公共服务透明供给。

　　扎实推进廉政建设。把阳光作为最好的防腐剂，把权力置于公众的有效监督之下，以更加坚定的信心、更加坚决的态度、更加有力的措施推进反腐倡廉，集中力量解决人民群众反映强烈的突出问题，深入推进工程建设等领域的专项治理，坚决纠正损害群众利益的不正之风，以反腐倡廉的实际成果保障发展、赢得民心。

　　各位代表！十堰正处在跨越式发展的关键时期，新的历史征程召唤着我们，新的宏伟目标激励着我们。让我们在市委的正确领导下，在市人大、政协的监督支持下，倍加珍惜千载难逢的历史机遇，倍加珍惜来之不易的发展环境，倍加珍惜政通人和的大好局面，乘势而上，奋力拼搏，为开创十堰美好明天而努力奋斗！
